# Cooperative catalysis of cellulose nanofiber and organocatalyst in direct aldol reactions

**DOI:** 10.1038/s41598-018-22350-5

**Published:** 2018-03-06

**Authors:** Kyohei Kanomata, Naoko Tatebayashi, Xin Habaki, Takuya Kitaoka

**Affiliations:** Department of Agro-Environmental Sciences, Graduated School of Bioresource and Bioenvironmental Sciences, Kyushu University, 6-10-1 Hakozaki, Higashi-ku, Fukuoka 812-8581 Japan

## Abstract

Cellulose nanofibers (CNFs) are finding a wide range of applications in the forthcoming sustainable society because of their carbon-neutral renewability and superior physicochemical properties. Here, we first show a cooperative organocatalysis by combining TEMPO-oxidized cellulose nanofiber (TOCN) and proline to enhance the catalytic efficiency in a direct aldol reaction. The yields of proline-catalyzed aldol products drastically increased in the presence of catalytically-inactive TOCN. This effect was also achieved by simply adding the TOCN to the reaction conditions where various proline analogues including structurally simple pyrrolidine and piperidine were used instead of proline. TOCN was superior to physically-pulverized CNF in the organocatalytic efficiency, and the nanofibrillation of cellulose microfibrils in reaction media was essential to induce the drastic enhancement in catalytic activity. The present finding will bring a new entry in the applications of CNFs, and open up a new phase in developing highly efficient molecular transformations in green chemical industries.

## Introduction

Cellulose is the key material to unlocking the gate toward a forthcoming sustainable society, because not only of its huge abundance and carbon-neutral renewability, but also its fascinating physicochemical properties, which are equivalent or sometimes superior to those of artificial and synthetic materials. Native cellulose, β-1,4-d-glucopyranose homo-polymer, forms highly crystalline structures with regular hierarchical assembly of extended chains in parallel alignment, leading to the construction of very fine nanofibers with 3–50 nm widths, especially those in wood cell walls. Therefore, cellulose nanofibers (CNFs) have attracted much attention both in academic and industrial circles, because of their well-defined architectures, which result in high mechanical strength like steel, low thermal expansion rate like quartz, high aspect ratios like carbon nanofibers, high transparency like plastics, and high chemical resistance like ceramics^1–3^. Thus, the applications of CNFs have been intensively studied for composite fillers^4^, electronic devices^5,6^, gas barrier films^7^, and various gels in wet and aero forms^8,9^. These applications have been aimed to design structural materials using the superior physical properties of CNFs.

The next-generation trends of applying CNFs have occupied much interest in catalytic transformations, through harnessing their characteristic and large surface areas via functional tuning. Serizawa *et al*. have reported hydrolysis of esters, monophosphates, and peptides in which acid-hydrolyzed cellulose nanowhiskers act as a catalyst, although the reactions require several days to proceed^10,11^. Metal nanoparticles like Pt, Pd, Ag, and Au deposited onto the surfaces of CNFs have been employed in various catalytic reactions^12^ such as hydrogenation of carbonyls^13^, reduction of 4-nitrophenol^14^, and carbon–carbon bond forming reactions^15^. Very fine and rigid CNFs are suitable for immobilization and exposure of metal nanocatalysts, demonstrating high catalytic performances.

**Figure 1:**
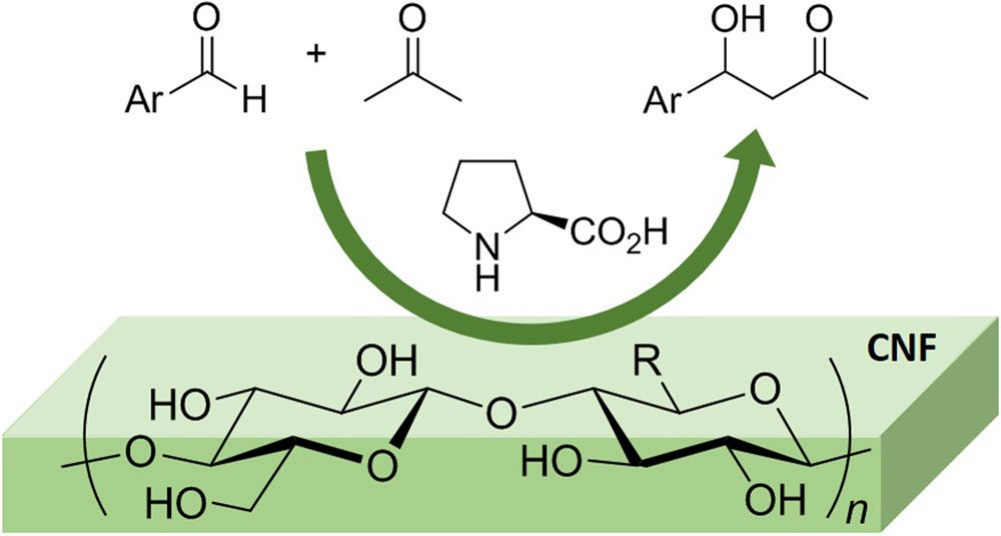
Schematic illustration of proline-catalyzed aldol reaction on cellulose nanofiber (CNF). R = CH_2_OH or COONa.

Organocatalysts have emerged as an alternative of rare metal catalysts in the past two decades, because of their chemically stable, functionally designable, and environmentally benign nature. Proline, a natural amino acid, has played a central role in various organocatalysis since pioneering work by List and co-workers^16,17^. Proline catalyzes the direct intermolecular aldol reactions between ketones and aldehydes to provide β-hydroxyketones, which are privileged structures in various pharmaceutical molecules. Proline, however, has been suffering from very low catalytic activities and poor solubility in common organic solvents, often resulting in extremely high catalyst loadings. Much effort has been made to improve the reaction efficiency, including the structural design of catalysts^18–20^, optimization of reaction conditions^21^, and employment of some additives^22–28^. During our challenge to develop catalytic systems by utilizing CNFs, we recorded an unexpected effect of CNFs on the catalytic efficiency of proline-mediated aldol reaction. Herein, we first report a unique combination of CNF and proline to enhance the efficiency of organocatalytic aldol reaction significantly (Fig. 1).

## Results and Discussion

Among various CNFs prepared by several different methods, 2,2,6,6-tetramethylpiperidine-1-oxyl (TEMPO)-oxidized cellulose nanofiber (TOCN) was focused on in this work for the initial screening because it provides a well-defined nanostructure with the narrowest fiber width (3–5 nm), and the largest specific surface area among those ever reported; carboxylate groups introduced to the surfaces of crystalline CNFs can facilitate disintegration of bundled nanofibers by electrostatic repulsion and osmotic effects in solvents^29^. Characterization of CNF and TOCN used in this study is shown in Figures S1–S3 in the Supplementary Information (SI). The aldol reaction of 4-nitrobenzaldehyde (4-NBA) with acetone was performed in the presence of proline (Table 1). The reaction efficiency was quite low in methanol, even when a large amount of proline (10 mol% against 4-NBA) was used, and the corresponding aldol adduct was obtained in a low yield (entry 1). By contrast, the addition of TOCN to the present reaction (equivalent weight to 4-NBA), significantly enhanced the reaction efficiency up to 86% yield (entry 2). TOCN alone did not promote the reaction at all (entry 3). These results evidently indicate that catalytically-inactive TOCN enhances the catalytic activity of proline in the aldol reaction. This is in sharp contrast to widely explored polymer-supported proline catalysts, which generally diminishes the catalytic activity^30^. The ratio of TOCN to 4-NBA was also investigated (entries 4–6). The yield was improved according to the increase of the amount of TOCN, and reached the maximum when the weight/weight ratio was 1:1 (entries 4, 5 and 2). Further increase of TOCN slightly diminished the reaction yield (entry 6). The conventional conditions of proline-catalyzed aldol reactions require considerably high catalyst loading (up to 30 mol%) to obtain the products in an acceptable yield^16^. Conversely, reducing the catalyst loading causes drastic decrease in the reaction efficiency^31^. On the other hand, our methodology succeeded in significantly reducing the catalyst loading to afford high yields under the mild and environmentally-benign reaction conditions. It is noteworthy that the present reaction can proceed in methanol, which is not a common solvent for aldol reactions catalyzed by proline and its derivatives. It is likely essential to employ suitable solvents for keeping the dispersion of TOCN stable, because acetonitrile and toluene with poor affinity for CNFs were less effective (see the SI for solvent screening; Table S1). Some additives, such as acids^22^, bases^22^, and water^23–25^ have been intensively studied for proline (and its derivatives) catalysis of aldol reactions in literature, and positive effects of water have been revealed. Detailed mechanistic studies suggested that water suppresses the formation of the off-cycle intermediate^23^, or in some cases, mediates a proton relay in a transition state^26^. Hydrogen bond donors such as diols^27^ and thioureas^28^ were also identified as effective additives to improve the solubility of proline in nonpolar organic solvents. TOCN used in this study possessed large amounts of carboxylates on the surface, which may potentially act as acidic and/or basic centers in organocatalysis; however, the carboxylate content of TOCNs had nearly no contribution to the reaction efficiency (see the SI; Table S2). In this study, the reaction using TOCN with lower carboxylate content (0.43 mmol/g-TOCN) proceeded similarly as compared to that with higher carboxylate content (1.68 mmol/g-TOCN). Therefore, our method is clearly different from these conventional systems, and the addition of TOCN disclosed here provides an entirely new approach to organocatalytic methodologies.

**Table 1:** Effects of TEMPO-oxidized cellulose nanofiber (TOCN) addition on proline-catalyzed aldol reaction of 4-nitrobenzaldehyde (4-NBA) with acetone.


Entry	(*S*)-Proline	TOCN/4-NBA ratio^**a**^	Yield (%)^**b**^
1	+	0	12^c^
2	+	1.0	86^d^
3	−	1.0	<3
4	+	0.3	18
5	+	0.5	26
6	+	2.0	72

Unless otherwise noted, the reaction was performed with 200 mg of 4-NBA (1.33 mmol), 200 mg of TOCN (COONa: 0.290 mmol), 8 mL of acetone (20 vol%), and 10 mol% of proline in methanol (32 mL). ^a^Weight/weight ratio of TOCN/4-NBA. ^b^Isolated yield. ^c^Enantiomeric ratio (e.r.) of the product was 69:31 (*R*:*S*), which was determined by chiral stationary phase supercritical fluid chromatography. ^d^e.r. = 55:45 (*R*:*S*).

With the prominent effect of TOCN demonstrated, the generality of catalysts was investigated. Here, the aldol reaction was performed for a series of proline analogues with or without TOCN (Table 2). The enhancement of the reaction rates was observed for (*R*)-proline as well as (*S*)-proline, suggesting that the chirality of proline does not affect the catalytic enhancement (entry 1). The reaction rates were also accelerated by adding TOCN with azetidinecarboxylic acid (entry 2) and tetrazole-type catalyst (entry 3). Furthermore, an effect was also observed to some extent with amide-type catalysts (entry 4) and even with pyrrolidine (entry 5) and piperidine (entry 6). The enhancement of the reaction rate is salient when there were acid functionalities in the catalyst molecules (entries 1–3), although they are not prerequisite for the present reaction system (entries 4–6). The acid functionalities are engaged in the stabilization of the transition state in proline-mediated aldol reactions^32,33^. Thus, the reaction with the catalysts bearing acid functionalities smoothly afforded the aldol product, whereas other catalysts without acid moieties provided several byproducts along with the desired aldol product, resulting in relatively low yields. Moreover, the yield remained low even in the presence of TOCN when pipecolic acid was used as the catalyst (entry 7). Pipecolic acid probably does not have a catalytic potency, regardless of the presence of TOCN.

**Table 2:** Aldol reactions in the presence of TEMPO-oxidized cellulose nanofiber (TOCN) using proline analogues as the catalyst.


Entry	Catalyst	Yield (%)^a^ TOCN(−)/(+)	Entry	Catalyst	Yield (%)^a^ TOCN(−)/(+)
1		8/83	5		20/63
2		4/72	6		10/57
3		8/78	7		<3/4
4		<3/16			

Unless otherwise noted, the reactions were performed with 300 mg of TOCN (COONa: 0.435 mmol), 300 mg of 4-nitrobenzaldehyde (1.99 mmol), 10 mL of acetone (20 vol%) and 10 mol% of catalyst in methanol (40 mL). ^a^Isolated yield.

We verified the effects of nanofibrillation of cellulose on the enhancement of catalytic efficiency (Table 3). Because the TOCN used in this study is a crystalline nanofiber densely bearing carboxylate groups as sodium salts on its surface (COONa: 1.45 mmol/g-TOCN, 21.8 mol% to 4-NBA in the present reaction conditions), the effects of nanofibrillation and carboxylate groups on the catalytic efficiency were examined independently. Thus, the addition of physically-nanofibrillated cellulose nanofiber prepared by an aqueous counter collision method^34^, which was free of carboxylate groups, also enhanced the catalytic efficiency, although prolonged reaction time was required to achieve high yield (entry 1). The effectiveness of physically-nanofibrillated CNF is lower compared with that of TOCN, even though the carboxylate-free CNF had high crystallinity (Figure S2) and nanodispersibility (Figure S3). This is probably because the fiber width of CNF (>10 nm) is thicker than that of TOCN (3–5 nm); hence, physically-nanofibrillated CNF could have a lower surface area than that of ultrathin TOCN (Figure S1). As for TOCN, the catalytic enhancement was very sensitive to the dispersibility of TOCN in methanol; gel-like aggregation of TOCN sometimes occurring during solvent-exchange procedure and/or dispersion in methanol completely lost the effect. These results strongly suggest that nanofibrillation of cellulose microfibrils is essential to enhance the catalytic activity. The addition of TEMPO-oxidized cellulose in a pulp fiber form without any fibrillation processing (TOC pulp; carboxylate content; 1.76 mmol/g-TOC pulp), which bears similar amount of carboxylate groups as the TOCN used in Table 1, did not improve the catalytic efficiency, and the yield remained low even after prolonged reaction time (entry 2). A slight increase in the yield can be attributed to the partial nanofibrillation of the surface of pulp fibers by the TEMPO oxidation. The effects of carboxylate groups were further studied; thus, the yield of the reaction with sodium acetate, whose molar amount was identical to that of carboxylate groups in TOCN, was as low as that without any additives (entry 3 vs Table 1, entry 1). Besides, as above described, TOCNs with different carboxylate contents of 0.43 and 1.68 mmol/g-TOCN did not show any significant difference in the reaction efficiency (see the SI; Table S2). Next, structural importance of crystalline nanofiber form was elucidated by comparing TOCN as a solid nanofiber and other polycarboxylates as a macromolecule. Neither sodium polyacrylate nor carboxymethylcellulose sodium salt, being adjusted to have the same amount of carboxylate groups as the TOCN used in Table 1, were effective in this reaction (entries 4 and 5). Thus, we assume that the characteristic nanoarchitectures of CNFs including TOCN must play a critical role in accelerating organocatalytic reactions. In one possible mechanism, some hydrophobic interaction between reactants and CNF surfaces would be involved in the catalytic reactions. The adsorption of aromatic compounds such as benzaldehyde to cellulose surfaces has widely been studied by thermodynamic analysis and molecular modeling^35,36^. In addition, it is well known that the aromatic residues of tyrosine and tryptophan of various glycohydrolases play an essential role in their strong binding to crystalline cellulose microfibrils^37,38^. Thus, the crystalline surfaces of CNFs possibly interacts with aromatic 4-NBA to increase its local concentration on the surface, resulting in accelerating the reaction. While many researchers have reported the effects of acidic and basic additives on aldol reactions^22,27,28^, our proposed reaction system would be completely different from the previous ones in the catalytic mechanism, although partial contribution of carboxylate groups cannot be ruled out. Further investigation is now underway in our laboratory to elucidate the evident effect of the crystalline surfaces of CNFs on organocatalytic behavior.

**Table 3:** Verification of nanofibrillation of cellulose on the enhancement of catalytic efficiency.


Entry	Additive	Yield (%)^a^
1^b^	Physically-nanofibrillated carboxylate-free CNF	78
2^c^	TOC pulp without nanofibrillation	32
3^c^	Sodium acetate	18
4^c^	Sodium polyacrylate	13
5^c^	Carboxymethylcellulose sodium salt	22

Unless otherwise noted, the reaction was performed with 200 mg of 4-nitrobenzaldehyde (1.33 mmol), 8 mL of acetone (20 vol%), and 10 mol% of (*S*)-proline in methanol (32 mL). ^a^Isolated yield. ^b^The reaction was performed for 24 h. ^c^The amount of carboxylate group was set identical at 0.290 mmol in the reaction system by adjusting the dosage of each additive.

## Conclusion

In summary, we show, for the first time, an unexpected nature of cellulose nanofibers to enhance significantly the catalytic activity in proline-mediated organocatalytic aldol reaction. Nanofibrillation of cellulose microfibrils is essential for promoting the present reaction system. The novel finding is a new entry in the applications of CNFs. Furthermore, this operationally simple methodology to enhance the catalytic activities brings alternative approaches in developing highly efficient catalytic transformations.

## Methods

### Materials

TEMPO-oxidized cellulose nanofiber (TOCN, carboxylate content; 1.45 mmol/g-TOCN) and never-dried sulfite pulp were kindly supplied by Nippon Paper Industries Co., Ltd. (Tokyo, Japan). Microcrystalline cellulose (Ceolus ST-100) was kindly supplied by Asahi Kasei Corp (Tokyo, Japan). Other Chemicals of laboratory grade were purchased from Wako Pure Chemical Industries Ltd. (Osaka, Japan) and used without further purification.

### Preparation of physically-nanofibrillated cellulose nanofiber

Microcrystalline cellulose (4.5 g; Ceolus ST-100, Asahi Kasei, Tokyo, Japan) was suspended in 300 mL of deionized water (1.5 wt%). The suspension was subjected to the aqueous counter collision method (245 MPa, 175 passes) using high-pressure water jet system equipped with a dual-nozzle chamber of 0.1 mm in diameter (Star Burst Labo, Sugino Machine Limited, Uozu, Japan). The obtained mixture was then centrifuged at 12,000 × *g* for 20 min to afford 0.6 wt% of cellulose nanofiber water suspension as a supernatant.

### Preparation of TEMPO-oxidized cellulose pulp without nanofibrillation

Never-dried softwood bleached sulfite pulp (31 wt%, 9.68 g; 3.0 g of dried form) was suspended in water (300 mL) containing TEMPO (48 mg) and sodium bromide (300 mg). TEMPO-mediated oxidation was started by adding sodium hypochlorite pentahydrate (4.94 g, 10 mmol per gram of cellulose) to the cellulose suspension. The pH of suspension was maintained to be 10 by adding 0.5 M NaOH with a pH stat for 4 h. the oxidation was quenched by adding ethanol (30 mL). The oxidized cellulose was thoroughly washed with water by centrifugation repeatedly at 4300 × *g* for 10 min (5 times). The carboxylate content of TEMPO-oxidized cellulose in a pulp fiber form (TOC pulp) was determined by conductometric titration (carboxylate content; 1.76 mmol/g-TOC pulp).

### Proline-catalyzed aldol reaction assisted by TOCN

Water suspension of TOCN (1.0 wt% suspended in water, 20 mL, 200 mg of cellulose) was mixed with acetone (30 mL), and the mixture was shaken several times, followed by centrifugation at 12,000 × *g* for 30 min. After the supernatant was removed, fresh acetone was added and the mixture was centrifuged again. This solvent-exchange treatment was repeated five times. The precipitate of TOCN was suspended in methanol (32 mL) and 4-NBA (200 mg, 1.32 mmol), acetone (3.0 mL), and (*S*)-proline (15.2 mg, 0.132 mmol, 10 mol% for 4-NBA) were added to the methanol suspension. The mixture was stirred at 40 °C for 4 h. The reaction was quenched with NH_4_Cl aq. and extracted with dichloromethane. After drying over MgSO_4_ and concentrating, the residue was purified by silica gel column chromatography to afford the product as a pale yellow solid. The product was characterized by ^1^H and ^13^C NMR spectra. The enantiomeric ratio was determined by supercritical fluid chromatography with a chiral stationary phase.

### 4-Hydroxy-4-(4-nitrophenyl)butan-2-one

Pale yellow solid; supercritical fluid chromatography analysis; AS-3 (CO_2_/IPA = 85/15, 1.0 mL/min, 254 nm, 40 °C), 0.84 (*R*), 1.13 (*S*) min; ^1^H NMR (CDCl_3_, 400 MHz): δ 2.22 (3 H, s), 2.81–2.89 (2 H, m), 3.56 (1 H, brs), 5.27 (1 H, dd, *J* = 5.6, 2.4 Hz), 7.54 (2 H, d, *J* = 6.0 Hz), 8.21 (2 H, d, *J* = 6.0 Hz); ^13^C NMR (CDCl_3_, 100.5 MHz): δ 30.6, 51.4, 68.8, 123.6, 126.3, 147.1, 150.0, 208.4 (see NMR spectra in the SI).

### Data availability statement

All the data generated and/or analyzed during the current study are included in this article and the Supplementary Information file, and are available from the corresponding author on reasonable request.

## Electronic supplementary material

Supplementary Information
