# Supplementary material for: Cooperative catalysis of cellulose nanofiber and organocatalyst in direct aldol reactions
Source: Sci Rep. 2018 Mar 6;8:4098. doi: 10.1038/s41598-018-22350-5 (PMC5840413; doi:10.1038/s41598-018-22350-5)
Supplement: Supplementary file 1 — Supplementary Information [file 41598_2018_22350_MOESM1_ESM.pdf]

Supplementary Information

Cooperative Catalysis of Cellulose Nanofiber and Organocatalyst in Direct Aldol Reactions

Kyohei Kanomata, Naoko Tatebayashi, Xin Habaki & Takuya Kitaoka*

Department of Agro-Environmental Sciences, Graduate School of Bioresource and Bioenvironmental Sciences, Kyushu University, 6-10-1 Hakozaki, Higashi-ku, Fukuoka 812-8581, Japan

Table of Contents

1. **Materials**
2. **Preparation of TOCN**
3. **TEM observation**
4. **XRD analysis**
5. **Birefringence observation**
6. **Proline-catalyzed aldol reactions assisted by TOCN**
7. **^{1}H and ^{13}C NMR spectra of the aldol product**
8. **References**

1. Materials

The catalytic reactions in Tables 1 and 2, and in Table S1 were carried out using 2,2,6,6-tetramethylpiperidine-1-oxyl (TEMPO)-oxidized cellulose nanofiber (TOCN, carboxylate content; 1.45 mmol/g-TOCN) supplied by Nippon Paper Industries Co., Ltd. (Tokyo, Japan). The reactions in Table S2 were performed using TOCN prepared in our laboratory from physically-nanofibrillated cellulose nanofiber (CNF, see the Section 2 in the Supplementary Information). Physically-nanofibrillated CNF used in Table 3, entry 1 was prepared from microcrystalline cellulose (Ceolus ST-100) supplied by Asahi Kasei Corp. (Tokyo, Japan) by aqueous counter collision method (see the Methods in the main text). The TOC pulp used in Table 3, entry 2 was prepared from never-dried sulfite pulp supplied by Nippon Paper Industries Co., Ltd. (Tokyo, Japan) by TEMPO oxidation (see the Methods in detail). Sodium polyacrylate (PAA, n = 22,000–70,000) and carboxymethylcellulose sodium salt (CMC, n = *ca.* 500) used in Table 3 were purchased from Wako Pure Chemical Industries Ltd. (Osaka, Japan) and Tokyo Chemical Industry Co., Ltd. (Tokyo, Japan), respectively, and used as received. The carboxylate contents of PAA and CMC were 11.7 and 2.74 mmol/g, respectively, which were determined by conductometric titration[1].

2. Preparation of TOCN

Two kinds of TOCNs with carboxylate contents of 0.43 and 1.68 mmol/g were prepared from physically-pulverized CNF (BiNFi-s WMa-10002, Sugino Machine Limited, Uozu, Japan). In brief, CNF (6.0 g by dry weight) were suspended in water (300 mL) containing TEMPO (96 mg) and sodium bromide (600 mg). TEMPO-mediated oxidation was started by adding 2.0 M aqueous sodium hypochlorite solution to the suspension. The pH of the suspension was maintained at 10 by step-wise addition of 0.5 M aqueous NaOH solution using an automatic pH titrator (Mitsubishi Chemical Analytech, Yamato, Japan). The oxidation was quenched by adding ethanol (6 mL) when the pH became constant. The oxidized CNF, *i.e.* TOCN, was thoroughly washed with 0.1 M aqueous HCl solution and then water by repeated centrifugation at 4,170 *g* for 10 min (three times). The TOCN was re-suspended in water and neutralized with 0.5 M aqueous NaOH solution until the pH was greater than 10. The suspension was then subjected to the aqueous counter collision method[2] at 245 MPa, using a high-pressure water-jet machine equipped with a dual-nozzle chamber having opposed nozzles of diameter 0.1 mm (Star Burst Labo, Sugino Machines Limited, Uozu, Japan). The carboxylate contents of the TOCNs were determined by conductometric titration[1]. The addition of 0.75 and 5.0 mmol of sodium hypochlorite per 1 g of cellulose (dry weight) afforded TOCNs with carboxylate contents of 0.43 and 1.68 mmol/g, respectively.

3. TEM observation

Transmission electron microscopy (TEM) was performed using a JEM-2100HCKM microscope (JEOL, Tokyo, Japan), operated at an accelerating voltage of 200 kV, at the Ultramicroscopy Research Center, Kyushu University, Japan. A 0.05% CNF dispersion (5 μL) was mounted on a glow-discharged carbon-coated Cu grid. The excess liquid was absorbed with a filter paper, and a negative staining reagent, 1% sodium phosphotungstate (5 μL), was dropped onto the sample. After standing for 5 min, the excess liquid was wiped with a filter paper. A drop of water (5 μL) was mounted on the sample grid, and then removed again with a filter paper after 3 min to wash away excess staining reagent. After air-drying, the samples such as CNF and TOCN on the grid were observed using the TEM apparatus in a bright-field mode.

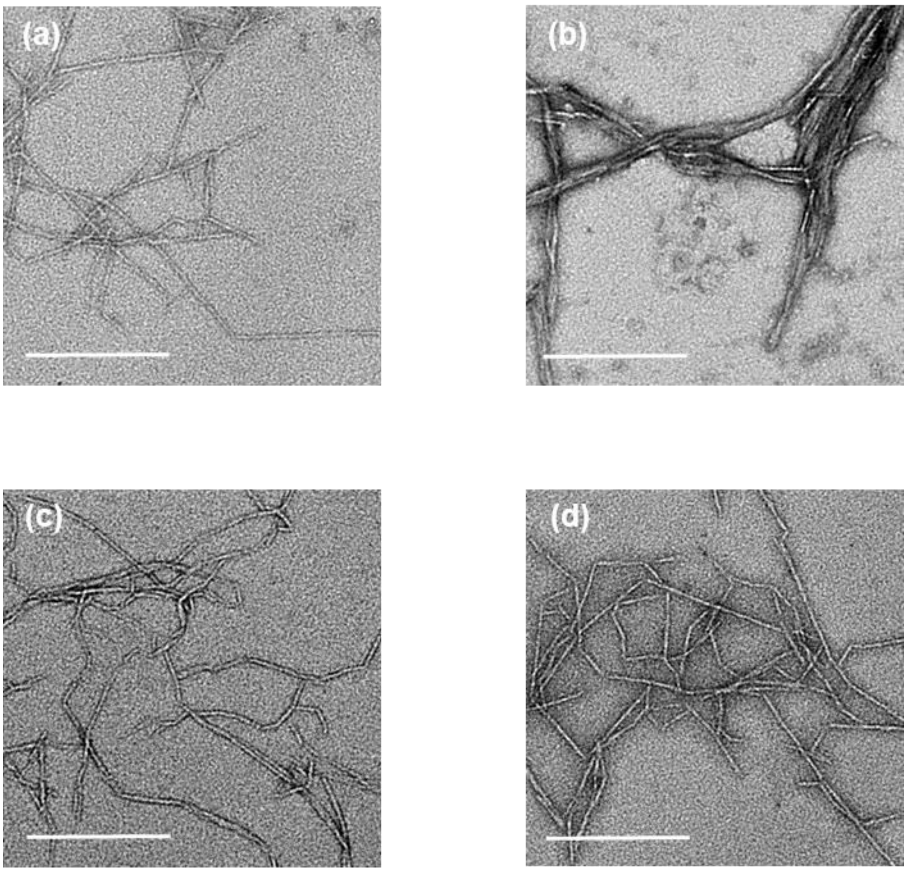

Figure S1. TEM images of (a) TOCN supplied by Nippon Paper Industries Co., Ltd.; (b) physically-nanofibrillated CNF prepared from Ceolus ST-100; (c) TOCN with carboxylate content of 0.43 mmol/g prepared from BiNFi-s; (d) TOCN with carboxylate content of 1.68 mmol/g prepared from BiNFi-s. Scale bars: 200 nm.

4. XRD analysis

X-Ray diffraction (XRD) patterns were recorded using an RINT 2000 (Rigaku, Tokyo, Japan) instrument with Ni-filtered Cu Kα radiation (λ = 0.1528 nm) at 40 kV and 20 mA. Scanning rate was set at 0.5° min^{-1} with 0.1° intervals. Samples were prepared by pressing freeze-dried CNF to make a pellet. The crystallinity index (CrI) was calculated using equation (1), based on the method reported by Segal and co-workers[3].

$$\mathrm{CrI}\ (\%) = [(I_{200} - I_{am})/I_{200}] \times 100 \qquad (1)$$

where I_{200} is the intensity at $2\theta = 22.5°$ and I_{am} is that of amorphous region at $2\theta = 18.7°$.

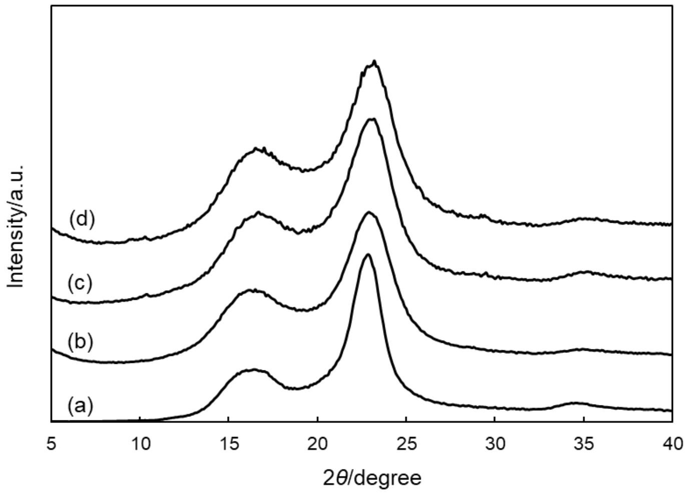

	CrI (%)
(a)	77.4
(b)	66.5
(c)	64.5
(d)	62.5

Figure S2. XRD patterns of (a) physically-nanofibrillated CNF prepared from Ceolus ST-100; (b) TOCN supplied by Nippon Paper; (c) TOCN with carboxylate content of 0.43 mmol/g prepared from BiNFi-s; (d) TOCN with carboxylate content of 1.68 mmol/g prepared from BiNFi-s.

5. Birefringence observation

Birefringence of 0.5% (w/v) CNF/water suspensions put between two crossed polarizers was observed at room temperature under stirring.

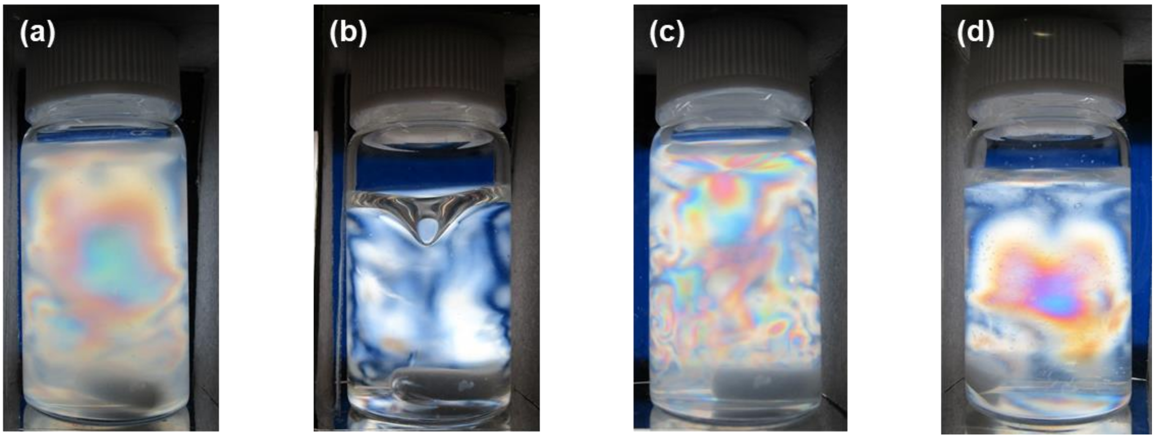

Figure S3. Birefringence images of (a) physically-nanofibrillated CNF prepared from Ceolus ST-100; (b) TOCN supplied by Nippon Paper; (c) TOCN with carboxylate content of 0.43 mmol/g prepared from BiNFi-s; (d) TOCN with carboxylate content of 1.68 mmol/g prepared from BiNFi-s.

6. Proline-catalyzed aldol reactions assisted by TOCN

Table S1. Solvent and temperature screening of proline-catalyzed aldol reactions assisted by TOCN[a]

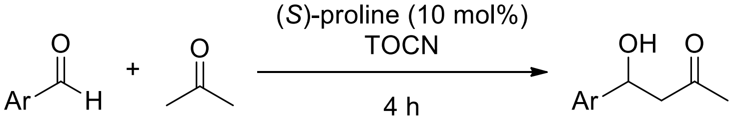

Ar = 4-$NO_2C_6H_4$-

Entry	Solvent	Temp./°C	Yield (%)[b]
1	MeOH	40	86 (12)
2	EtOH	40	46
3	DMSO	40	67 (35)
4	DMF	40	39
5	CH_3CN	40	12
6	Toluene	40	<3
7[c]	MeOH	25	76
8	MeOH	50	79

[a] The reactions were carried out with 200 mg of TOCN, 200 mg of 4-NBA (1.33 mmol), 8 mL of acetone (20 vol%), and 10 mol% of (*S*)-proline in each solvent (32 mL). [b] Isolated yield. The yields without TOCN are listed in parentheses. [c] The reaction was performed for 8 h.

Table S2. Proline-catalyzed aldol reactions using TOCNs with different carboxylate contents[a]

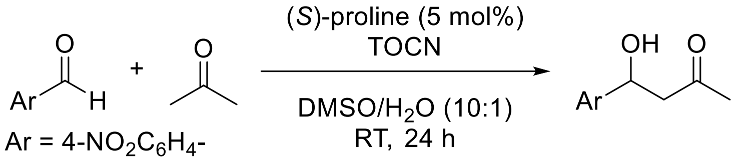

Carboxylate content (mmol/g-TOCN)	Yield (%)[b]
0.43	97
1.68	97

[a] The reactions were carried out with 100 mg of TOCN, 300 mg of 4-NBA (1.99 mmol), 10 mL of acetone, and 5 mol% of (*S*)-proline in 10/1 (v/v) mixture of DMSO and water (40 mL).
[b] Isolated yield.

Both TOCNs with carboxylate contents of 0.43 and 1.68 mmol/g-TOCN showed ultrathin, individual microfibrils in the TEM images (Figure S1) and clear birefringence (Figure S2). These results suggest that each nanodispersibility closely related to specific surface area is comparable for both TOCNs.

7. ^{1}H and ^{13}C NMR spectra of the aldol product

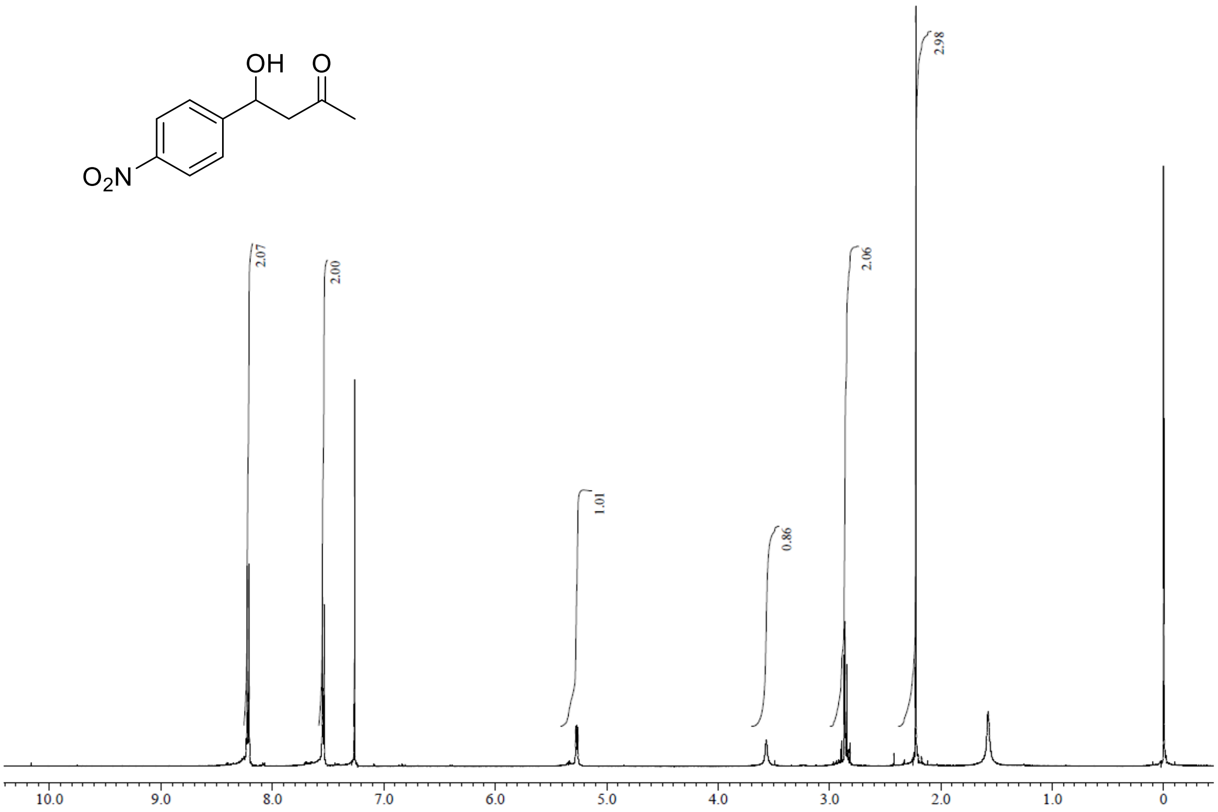

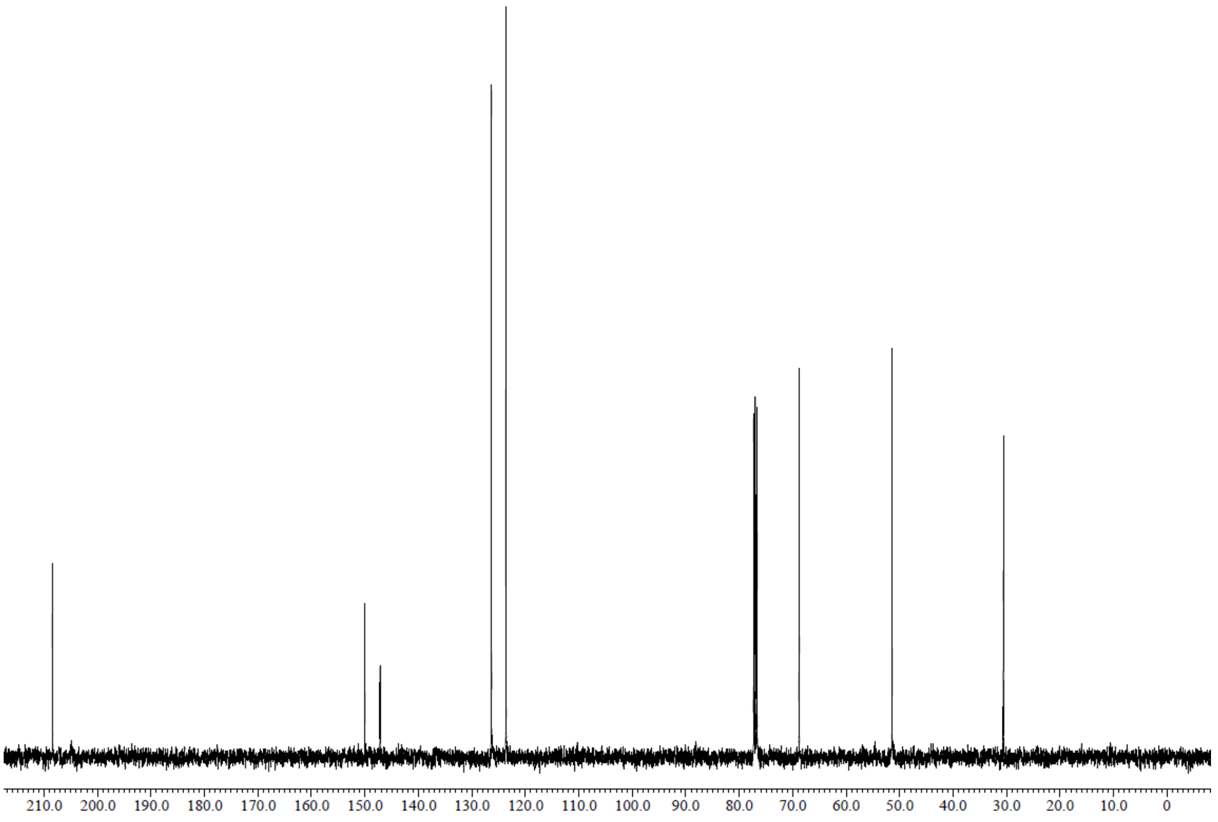

8. References

(1) Saito, T. & Isogai, A. TEMPO-Mediated Oxidation of Native Cellulose. The Effect of Oxidation Conditions on Chemical and Crystal Structures of the Water-Insoluble Fractions. *Biomacromolecules* **5**, 1983–1989 (2004).

(2) Kondo, T., Kose, R., Naito, H. & Kasai, W. Aqueous Counter Collision Using Paired Water Jets as a Novel Means of Preparing Bio-nanofibers. *Carbohydr. Polym.* **112**, 284–290 (2014).

(3) Segal, L., Creely, J. J., Martin, A. E. & Conrad, C. M. An Empirical Method for Estimating the Degree of Crystallinity of Native Cellulose Using the X-Ray Diffractometer. *Text. Res. J.* **29**, 786–794 (1956).
